# Sensor Prototype to Evaluate the Contact Force in Measuring with Coordinate Measuring Arms

**DOI:** 10.3390/s150613242

**Published:** 2015-06-05

**Authors:** Eduardo Cuesta, Alejandro Telenti, Hector Patiño, Daniel González-Madruga, Susana Martínez-Pellitero

**Affiliations:** 1 Department of Manufacturing Engineering, University of Oviedo, Campus de Gijón, Asturias 33203, Spain; E-Mails: aleteroso@gmail.com (A.T.); psanchezhector@gmail.com (H.P.); 2 Department of Mechanical, Informatics and Aeroespatiale Engineering, University of León, Campus de Vegazana, León 24071, Spain; E-Mails: danimadru@gmail.com (D.G.-M.); susana.martinez@unileon.es (S.M.-P.)

**Keywords:** coordinate measuring arm (AACMM; CMA), contact force sensor, probing control

## Abstract

This paper describes the design, development and evaluation tests of an integrated force sensor prototype for portable Coordinate Measuring Arms (CMAs or AACMMs). The development is based on the use of strain gauges located on the surface of the CMAs’ hard probe. The strain gauges as well as their cables and connectors have been protected with a custom case, made by Additive Manufacturing techniques (Polyjet 3D). The same method has been selected to manufacture an ergonomic handle that includes trigger mechanics and the electronic components required for synchronizing the trigger signal when probing occurs. The paper also describes the monitoring software that reads the signals in real time, the calibration procedure of the prototype and the validation tests oriented towards increasing knowledge of the forces employed in manual probing. Several experiments read and record the force in real time comparing different ways of probing (discontinuous and continuous contact) and measuring different types of geometric features, from single planes to exterior cylinders, cones, or spheres, through interior features. The probing force is separated into two components allowing the influence of these strategies in probe deformation to be known. The final goal of this research is to improve the probing technique, for example by using an operator training programme, allowing extra-force peaks and bad contacts to be minimized or just to avoid bad measurements.

## 1. Introduction

Recent developments in portable coordinate measuring arms (CMAs or AACMMs) have brought coordinate metrology closer to production, at the same time introduced higher accuracy than any other instrument has had before. CMAs have been designed as portable, versatile and flexible machines with the capacity to fulfil Geometric Dimensioning & Tolerancing (GD&T) tasks though coordinate measurement concepts. An optical scanner can also be attached to enable reverse engineering by non-contact measurement. The relatively lesser importance of accuracy in reverse engineering is, in practice, compensated for by the ease of measurement, speed, capacity to scan complex geometries, measurement strategy adaptability and point density among others. In any event, CMAs are easily integrated into a production environment without significant training in time terms.

CMAs are typically used for contact measurements in short measurement campaigns having few measurands and few workpieces. For instance, they are used for the GD&T quality control of assemblies [1] with welded or screwed plates and GD&T verification in the field, either at a tool machine table or directly at a production work station as in prototype manufacturing. CMAs are most suitable for companies needing fast measurements in different places, short measurement campaigns or verification of a few relevant measurements and dimensional or geometric tolerances. It is also common to find CMAs used in reverse engineering combined with dimensional metrology to determine the reference of complex geometries to simple geometries.

The relevance of CMA measurements is high since their range of uncertainty and flexibility cannot be easily covered by other equipment. High impact on the project costs or duration are avoided by using CMAs in different places and for different measurement tasks, as in the development of prototypes or assemblies in the field.

The reliability and traceability of CMAs cannot be evaluated in the same way as for Coordinate Measuring Machines (CMMs) due to the differences in the kinematic structure and usage. Specific standards have been developed for CMAs (ASME B89.4.22, [2], VDI/VDE 2617 [3] and the draft standard ISO 10360.12 [4]), but their reliability always requires elements, such as the probe type, the use of rotary encoders or the kinematic structure among others [5,6,7,8], to be taken into account..

CMM traceability has been widely studied and it can be easily guaranteed for common measurements, even under dynamic conditions [9]. Taking into account the automatic CMM point collection procedure, and the control of the influential factors (control of temperature, relative humidity, cleanliness…), it is reasonable that CMMs achieve even sub-micrometre uncertainties for small and medium volumes. The much improved uncertainty of CMMs against CMAs has been used to compare both systems and estimate the capacity of CMAs [10,11]. These works show the importance of the measured feature and the operator factor [12]. High variability in the measurement is found when different contact forces and CMA orientations are applied during the measurement.

This work describes in detail the development of a prototype sensor designed to characterize the measurement force during contact, the different operators, the measurement strategies and the probe geometry. Improvements and measurement techniques are proposed to enhance CMA reliability.

## 2. Knowing the Probing Force on CMAs. Previous Surveys and Improvement Proposals

**Figure 1:**
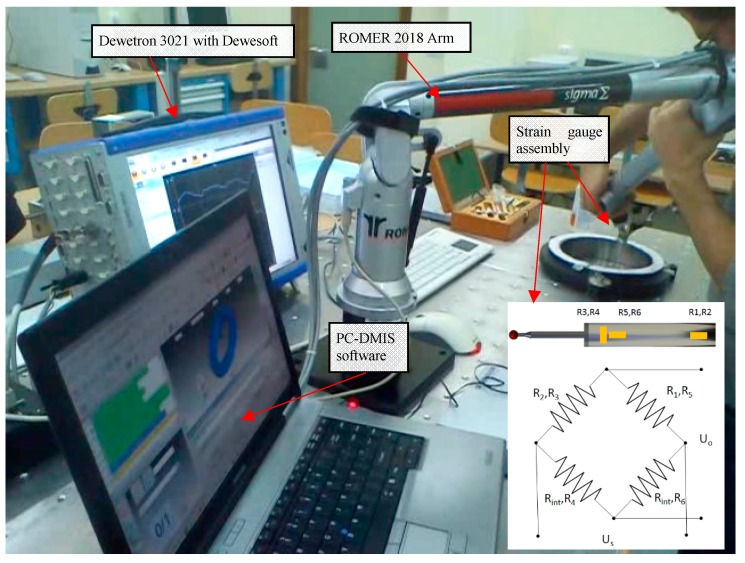
A view of the previous force gauge arrangement measuring a master ring. The configuration of the bridge circuit uses six strain gauges.

CMA technological evolution has reduced their weight by using light materials, increased their autonomy and probe rigidity, and improved their overall ergonomics, but has neglected the actual operator skill, which is ultimately responsible for the applied force and the final probing. Changes in contact force when measuring with CMAs are expected [13,14], with significant changes for one operator and among operators. Force parameters are also highly affected by the measured feature [11,15]. These changes cause a significant loss of CMA reliability in comparison with their potential performance. Recent investigations suggest that the monitoring of force (magnitude and direction) will allow the contact force to be controlled and its effects on the measurement accuracy to be reduced [16,17]. In this work, the human factor or the operator is highlighted as a significant uncertainty contributor for contact measurement. A primitive force sensor based on strain gauges mounted on the probe (Figure 1) has been developed [16].

It shows the relevance of the operator and the force in cylindrical geometries and concluded the necessity to study these factors. The contact force was quantified in a range of 0.5 N to 5 N with significant differences between operators (in force magnitude and direction). It also shows that error measurements are highly correlated with the contact measurement. The compensation for these factors and the deformation of the probe are key points in further study of the contact force in CMAs.

The limitations of the primitive sensor and our increased knowledge about the importance of force in CMA, measurement strategies, metrological analysis of the results and signal processing have led to the design and manufacture of a completely new sensor with the capacity to acquire relevant data in order to improve CMA performance to the maximum. It is necessary to design a customized housing integrated with the current CMA, incorporating an improved and protected strain gauge, an electrical synchronization circuit and a trigger mechanism to activate the signal at an exact measurement time. A final requirement is that these improvements have to be carried out at a reduced cost due to the limited economic annual allocation for the project. The reduced cost of the solution also gives an idea of its suitability for the manufacturer’s other CMA models and also its plausible integration into other commercial CMAs.

The new sensor is ergonomically integrated; therefore, the sensor does not alter the operator behaviour. Force signals are fully synchronized with point acquisition by the CMA software. In this method, outline data processing to determine the contact point is not necessary, providing only relevant information for the force analysis. This is particularly useful when different strategies are evaluated. Signal noise is reduced by the integration of the system including the fixed cable position and protecting the sensor itself. The final prototype could be assembled and disassembled without compromising the CMA structure or affecting its correct operation, avoiding any CMA damage or even new calibration. In fact, the new sensor is developed for a Sigma 2018 unit (ROMER^®^, Hexagon Metrology, Montoire, France) as an external attachable accessory (Table 1). As a result, this work presents the design, manufacture (by additive techniques and other processes), system adjustment, calibration and validation. Finally, the system has been used to study the contact force of the same operator and of different features and different operators.

**Table 1:** Coordinate Measuring Arm features.

Coordinate Measuring Arm Data	Specifications
Manufacturer—model	ROMER Sigma Arm 2018
Measuring range	1800 mm (spherical diameter)
Repeatability sphere test	±0.025 mm
Repeatability cone test	±0.032 mm
Length accuracy	±0.044 mm
Weight	5.2 kg/11.5 lbs

## 3. Development of an Integrated Force Sensor Prototype

The new sensor consists of the force sensor gauge arrangement glued to the stylus of the hard probe (Section 3.1) and the signal synchronization system located between the sensor and the CMA wrist (Section 3.2).

### 3.1. Force Sensor—Mounting and Integration

**Figure 2:**
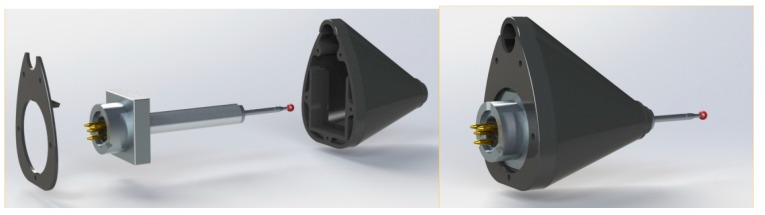
CAD detail design for the gauges’ protective housing.

Contact force values are obtained by strain gauge bridges which allow the deformation in the probe stylus [16,17] to be measured. A total of six strain gauges (R_1_, … R_6_) were used in two strain gauge bridges to measure pure axial force and bending force on the probe. R_3_, R_4_, R_5_ and R_6_ are 1-XY11-1.5/350 (HBM Inc., Marlborough MA, USA) and use axial force measuring with a full Wheatstone-bridge circuit, while R_1_ and R_2_ are 1-LY11-1.5/350 (HBM Inc., Marlborough MA, USA) and use bending force measuring with a half bridge circuit completed by the internal resistance (R_int_) provided by the data acquisition equipment (Dewetron 3021^®^, Dewetron GmbH, Grambach, Austria). The sensor was implemented on a new rigid probe (66 mm long, 10 mm in diameter, with a ruby ball of 4 mm in diameter). The output signals sent from the bridges are collected with a capture frequency of 1000 Hz. Both signals, axial and bending force, are detected and processed individually using two separated input channels in the acquisition system. The software of this instrument (Dewesoft^®^) allows configuration of the signals and the undertaking of a primary process that consists of adjusting the signals to specific levels in order to match the zero signal with the zero load and the maximum signal with the maximum load (which was tested and calibrated up to 6 N). The physical stability of the sensor and the stability of the signal (without disturbances generated by wire movement during measurement) have been ensured thanks to a self-protecting structure that fixes all the internal components during measurement. Rapid prototype techniques (Polyjet 3D Additive Manufacturing technology) were used to manufacture the protective gauge housing (Figure 2) as well as the handle and some internal parts of the prototype.

**Figure 3:**
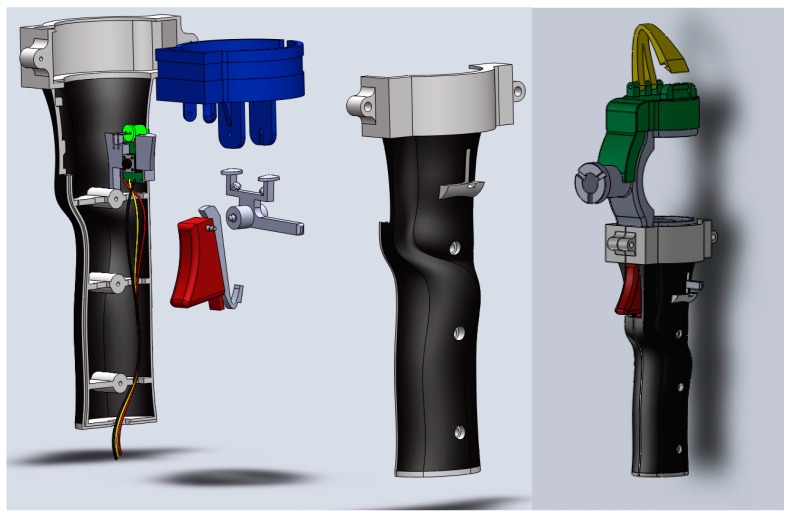
Final design of the handle with the synchronization system.

**Figure 4:**
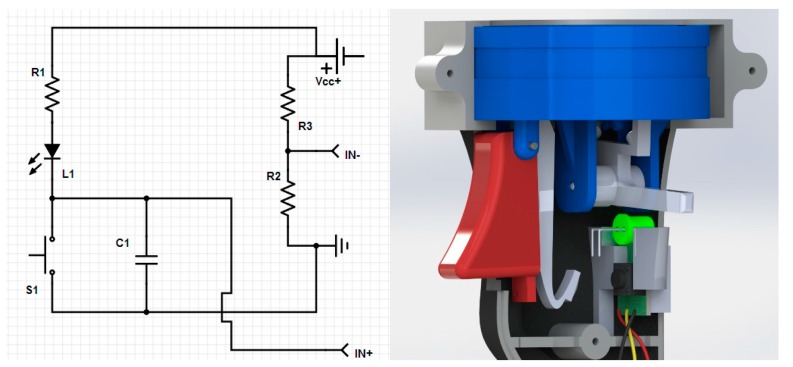
(**Left**) Electrical scheme for the triggering sensor; (**Right**) Details of the triggering mechanism.

**Figure 5:**
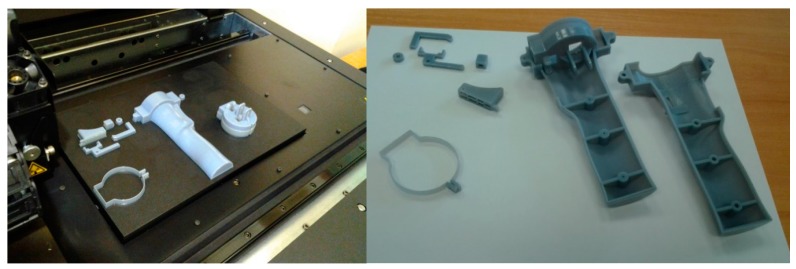
(**Left**) Synchronization system components manufacturing; (**Right**) Cleaned parts.

During the design stage, the manufacturing system was always taken into account. By taking advantage of rapid manufacturing technology, the part could be designed without considering clamping locations or the typical geometrical constraints imposed by the removal machining processes. Other design aspects, such as the constant thickness, the relief angles or the location of the injection points, usually considered in manufacturing plastic parts by injection, were not a concern either. This lack of constraint in the design applies to the housing for the gauges, the internal elements and the handle, shown in the following sections (Figure 3, Figure 4 and Figure 5).

### 3.2. Synchronization System

The synchronization system consists of an ergonomic handle and the system to synchronize force and point collection signals, including a point trigger mechanism and the circuit. The new handle has two parts, which contain all the elements (mechanical and electrical) allowing the integration and synchronization of the new sensor without losing any characteristic of the CMA. Typically when controlling a CMA, the operator positions the probe tip on the surface to be measured and the collected point is determined by pressing a button in the original handle. The design of the mechanism maintains it at the collection point. By pressing the new trigger, activation signals and the CMA point collection mechanism are simultaneously activated. Secondary functions of the original handle, such as deleting or accepting points, are also possible with the new system.

The handle consists of two twin parts that, when assembled, provide an ergonomic support for the sensor and offer enough protection to the rest of the components (Figure 3). It also contains internal structures to increase the rigidity of the handle. The triggering mechanism is supported by the handle providing full integration with the CMA hardware. On the back part of the handle, a green LED has been mounted so the triggering can be controlled visually by the operator. The system wires are fixed to a new mechanism (green and yellow parts on the right in Figure 3) by the handle so their movements do not produce instability, disturbance or noise in the signals.

An electronic circuit (PCB) was designed and implemented to detect the contact time (Figure 4, Left). It was connected to another channel of the data acquisition system. A total of three signals are collected: point coordinates by the CMA software, force (bending and axial) and the contact time by the data acquisition system. The circuit was miniaturized and integrated into the sensor, being activated by a lever (Figure 4, Right). With this system, the exact time of force reading is obtained, so only relevant information about force values is filtered and collected. It could be set so that this system can be easily adapted to other commercial CMAs, materializing in a new generation of CMA probe sensors.

## 4. Manufacturing and Global Assembly of the Prototype

As mentioned above, rapid prototype techniques were used to manufacture most of the parts: the sensor’s protective housing and the elements of the new handle, specifically Polyjet 3D AM technology. This technology consists of jetting layers of an acrylic-based photopolymer onto a flat surface. After each jetting cycle, each layer is cured with UV radiation, so that the material solidifies and additional layers can be subsequently stacked in the Z direction. Rapid prototype techniques allow the manufacture of custom 3D solutions not achievable with conventional manufacturing techniques. In particular, an Object 30 machine (Stratasys, Eden Prairie, MN, USA) was used. The model material was a Stratasys RGD240 photopolymer, while support material was a Stratasys FullCure 705 photopolymer. Layer thickness for this material was 28 µm. Resolution, in both the X and Y directions of the 3D printer, was up to 600 dpi (approximately 42 µm). The part accuracy was sufficient due to all the specimen geometry being designed using SolidWork^®^ software and converted to STL file format (0.001 mm conversion tolerance). Figure 5 shows details of the parts after manufacturing in the 3D printer and the same parts ready to be assembled on the CMA.

**Figure 6:**
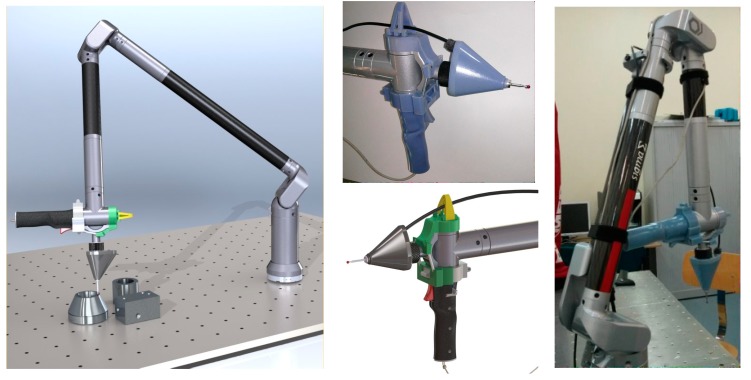
Details of the final sensor device, from concept to the real model.

Figure 6 shows the sensor design and the sensor mounted on the CMA. The sensor is completely integrated with the CMA geometry and software allowing ergonomic control of the CMA without any change in its performance.

The economic requirement to be a low cost prototype has prevented a more optimized solution, *i.e.*, with smaller parts, triaxial gauges, more resistant and durable materials, *etc.* Such improvements are left to the manufacturers with the hope that future CMA generations have this “probing force control” technology by default or at least as a modular option.

## 5. Calibration and Validation Tests

**Figure 7:**
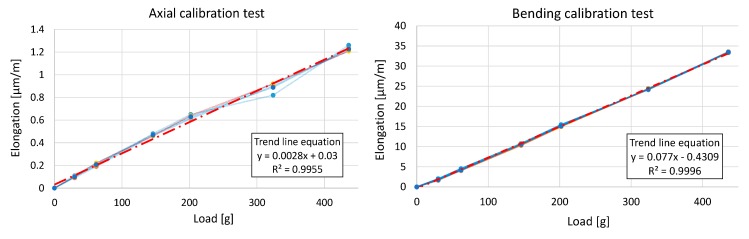
Sensor calibration tests: (**Left**) for axial loadings; (**Right**) for bending loadings.

Once the sensor had been implemented with the three signals (axial force, bending force and triggering signal), an application to monitor the signals was developed and the system was calibrated and validated. The calibration process consists of using standard weighs (M1 class in accordance with OIML R111-1) to find the relationship between the collected signal and the force on the probe. Weights of 0 to 1000 g were used, in accordance with the force range (and the maximum value reached) from previous studies. The weights simulate axial and bending forces on the probe, respectively, in separate tests. First, several standard weights, from 10 to 1000 g, were located (hung) in a pure axial position. This test implies ten repetitions of “increasing” and “decreasing” loading cycles in order to obtain the axial behaviour curve and its uncertainty (Figure 7, Left). Later, the same standard weights and the same cycles were repeated, but now located (again hung) in a pure bending position to obtain and calibrate the bending behaviour curve (Figure 7, Right). As can be seen in Figure 7, the response of the sensor signal was found to be linear to this range given a good grade of approximation for both components.

In fact, the expanded uncertainty for both force components was calculated using a GUM budget (*JCGM 100:2008-Guide to the Expression of Uncertainty in Measurement*) in accordance with previous studies [17]. This budget incorporates the contributions of several items (temperature, resolution, hysteresis, *etc.*) similar to the case of calibrating load cells and balances. With this procedure, expanded uncertainties (95% confidence level, k = 2) of 0.06 N (axial) and 0.11 N (bending) were respectively obtained. When the usual human force oscillates between 1 N and 6 N, these values represent a percentage between 1 and 6 in the axial case and between 1.83 and 11 in the worst case, bending. In addition, the higher the probing forces, the lower these percentages. So, for both components of force, these values indicate the successful manufacturing and calibration of the developed acquisition arrangement.

## 6. Experimentation and Results

**Figure 8:**
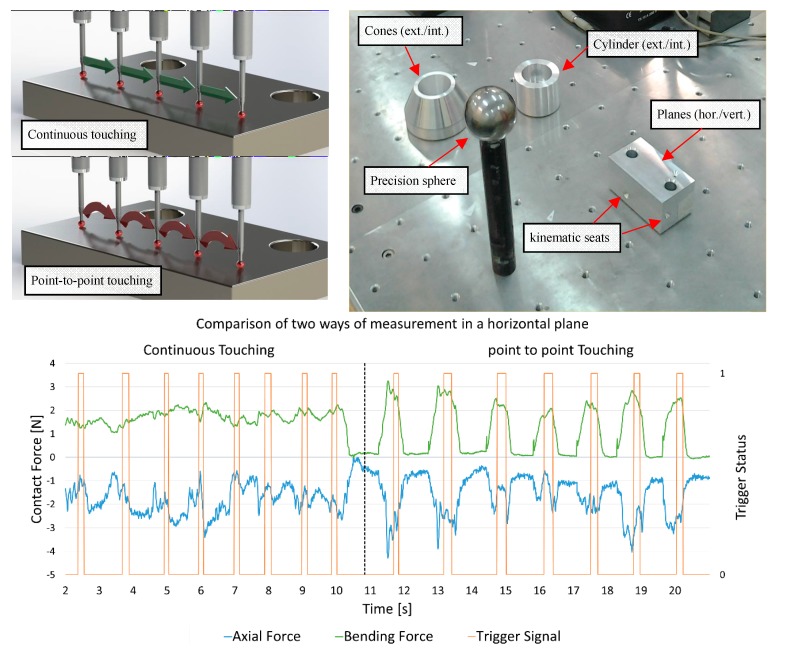
(**Top left**) two measurement methods; (**Top right**) test set-up; (**Bottom**) graphs obtained with the sensor: axial force, bending force and triggering signal.

**Figure 9:**
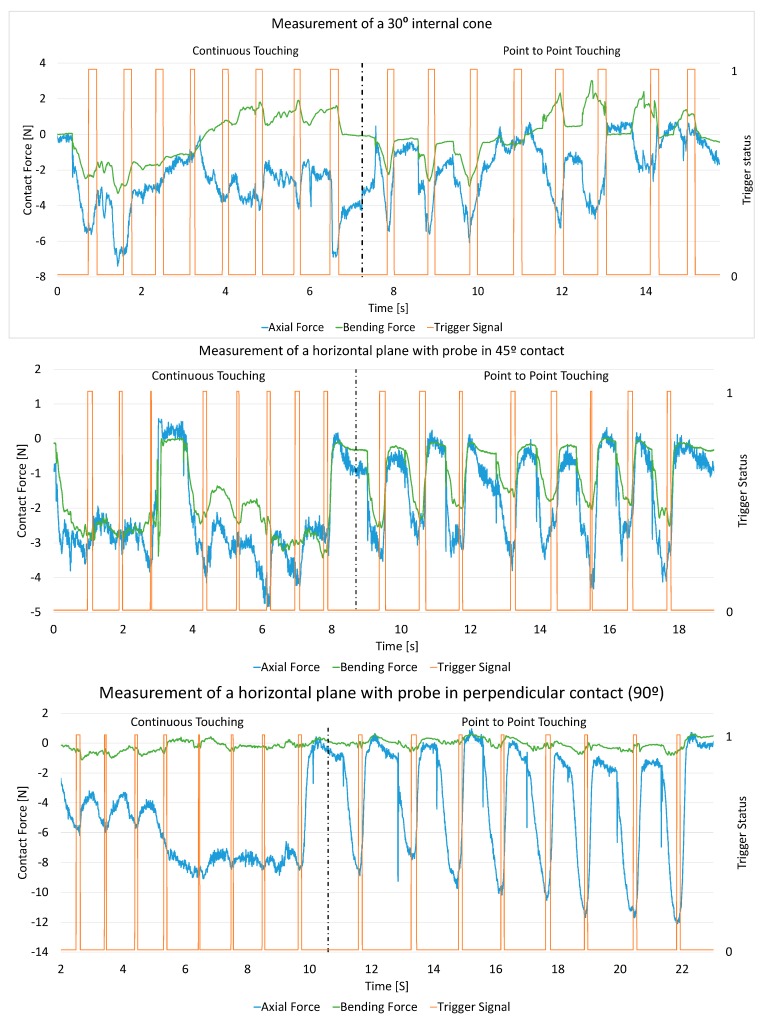

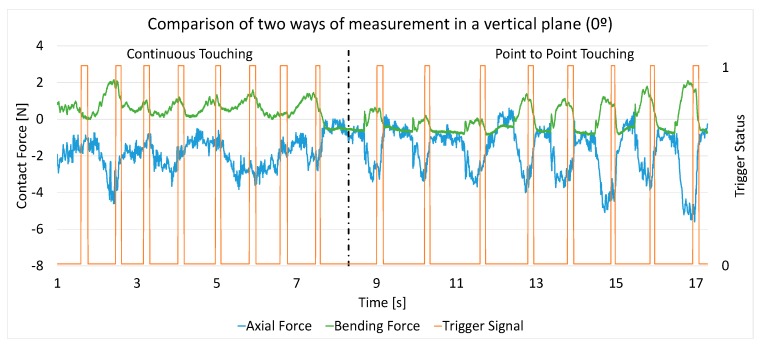
Examples for force reading when measuring different workpieces: continuous touching (left curves) and point-to-point touching (right curves).

Once the raw signal of the elongation gauges (in µm/m) were transformed into actual force measurements (thanks to the calibration test procedure), the signal was finally processed in Matlab^®^ and saved in MS Excel format in order to process the data and analyse the CMA performance. Similar graphs to Figure 8 and Figure 9 were obtained. The triggering force signal highlights the contact force applied when collecting points by the CMA.

Figure 8 shows the results for the measurement of a plane with eight points and two different measurement techniques. The first technique consists of measuring eight points while maintaining the contact between the part and the probe during the whole measurement (similar to scanning probes for CMM) and the second consists of measuring the eight points releasing the contact between the points (Figure 8, Left and Right, respectively). In the second, called “point-to-point touching” or discrete contact, the probe contacts the workpiece surface only to take the measurement of each of the eight points. The green signal represents the bending force while the blue signal represents the axial force. For this signal, negative values correspond to compression. The triggering signals, orange signals, indicate the exact time that the trigger button is pressed (vertical lines).

These graphs show the actual forces on the probe which seem to be similar in magnitude but the deformations caused by them are quite different. In previous studies [16,17], and using both this gauge arrangement and with FEM method validation, the deformations of the probe stylus were found to be very different depending on the direction of the force. If the force is in a pure axial direction, it generates ten times less deformation than in a pure bending direction, so although Figure 8 only shows the magnitude of the force, obviously the values—and shape—of the bending curve (in green) are very much more critical than the axial (blue curve). This is why high values of the bending force are always a critical parameter and should be controlled somehow.

Once the sensor was calibrated and this application developed, the test was performed on other features. These features were: cylinders (internal and external), cones (internal and external), horizontal and vertical planes and a precision sphere. The external and internal cylinder diameters are 60 mm and 50 mm, respectively. The cylinder height is larger than 36 mm for proper measurement. Cones have a diameter from 84 mm (ext.) to 55 mm (int.) with external and internal angles of 45° and 30°, respectively. The planes are contained in a prismatic workpiece with approximate dimensions of 50 × 50 mm with kinematic seats (conic holes where the sphere tip of the probe can fit). The sphere is made of tungsten carbide and has a diameter of 50 mm. The dimensions of the features were selected for comfortable and accessible measurement. The maximum form error for the features is 0.010 mm. The measurements were recorded in a laboratory where the temperature is controlled (20 ± 1 °C).

The test consists of the measurement of the features with the two measurement strategies and with several probe orientations and operators. For each workpiece, force and triggering graphs were obtained (Figure 9). For each graph, the left part shows the results for continuous touching and the right part shows the results for the point-to-point measuring strategy.

For instance, in the upper graph of Figure 9, the measurement of a 30° internal cone is represented. On its left side a green curve can be seen with high values of the bending force where the continuous touching strategy was in operation. On the right side of this upper graph, the point-to-point strategy was used. Here the operator makes some punctual force values, some of which could be higher than in continuous mode. Despite this fact, the average force value remains at a lower level than in the continuous strategy. This is because the more comfortable measuring position of the continuous mode, easily leads to an excess of contact force, giving a high average force. So in this case, measuring the feature in a safe way, using the point-to-point strategy, is suggested as it fosters careful probing. In summary, with regard to cone features, unless careful probing in continuous mode is undertaken, the discrete (point-to-point) probing strategy is preferable.

With regard to the planes, measurements at approximately 90° (quasi-perpendicular), 45° and 0° (quasi-parallel) were taken. Significant differences were found during the measurements. The second and third graphs in Figure 9 show that the operator exerted similar force, that force is easier to control when continuous measurements are applied and that contact in point-to-point produces high peaks of force.

The last graph in Figure 9 shows the force readings for the horizontal plane when measuring with the probe perpendicular and parallel to the surface. In the first case, there is almost no bending force, the axial force being the relevant force. Continuous measurement is preferred at 5 N of axial compression, while for point-to-point it can reach up to 10 N. When a vertical plane is measured parallel to the surface, continuous measurement is also preferred although it is not as clear as in the previous case. In this case, excess axial and bending forces are avoided by continuous measurement. Continuous measurement was not preferred by the manufacturers since it could cause difficulties in point acquisition; however, current CMA models emit a sound when the point is not correctly taken.

Kinematic seat (conic hole) measurement is one of the most interesting tests that can be performed in order to test the CMA measurement status in a quickly and easily way. When the sphere of a CMA probe fits into a conic hole, the probe is theoretically positioned always in the same spatial position because it is supported by the same contact circle (intersection between the sphere and cone), so repeatability can be studied. Also, some studies use several kinematic holes that define a virtual circle (three points) or a virtual sphere (four points) with no form error. The methodology is used to check CMA performance quickly [18,19,20]. There is also a test proposed by the standards [2,3] where kinematic seats are used to study the repeatability of the measurement of the same point with several positions of the CMA joints, the single point articulation test (SPAT).

**Figure 10:**
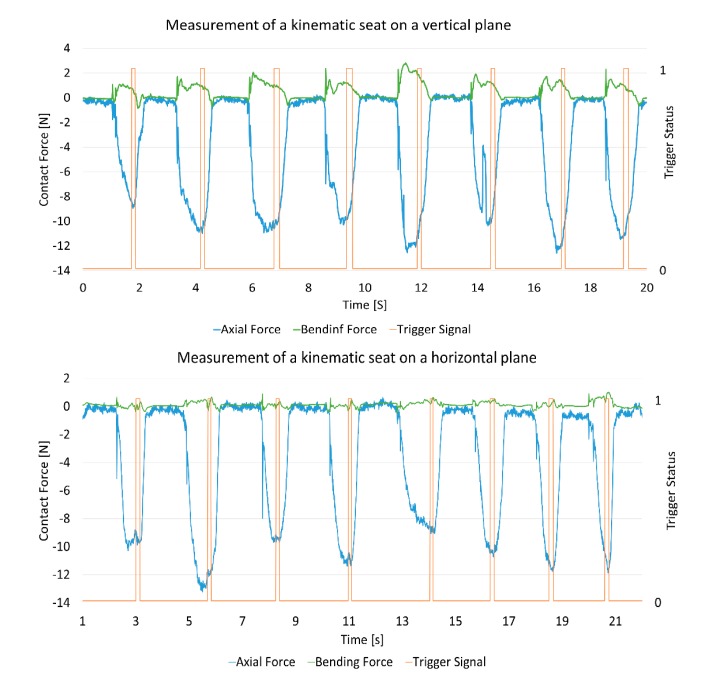
Examples of force readings when measuring kinematic seats. Trigger, axial and bending curves for (**Top**) vertical plane and (**Bottom**) horizontal plane.

The graphs in Figure 10 show the force signals for eight kinematic seat measurements. In the first graph, a kinematic seat located on a vertical plane is measured and the second corresponds to a kinematic seat located on a horizontal plane. Both graphs show high values of axial force, up to 13 N, being an extreme case for CMA measurement. In the case of the horizontal plane, the forces are always higher than 9 N, due to the force applied by the operator and the probe and CMA’s own weight. This test indicates that the forces applied are much higher than in the measurement of common features, such as cylinders or planes, with values up to three times the force of other features.

**Figure 11:**
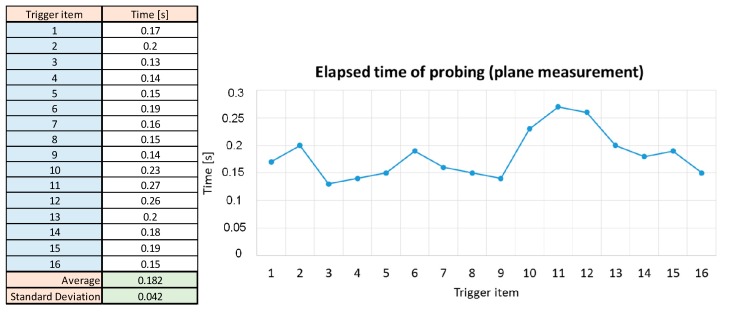
Duration time for triggering signal.

The synchronization of this sensor also allows the duration of the triggering time to be studied. The main objective of this survey is to study the homogeneity of the duration among operators. These data correspond to the horizontal part of the triggering signal. These values were collected and an average duration of 0.182 s was found, Figure 11. This was constant for all operators.

Regarding the range of force values, it must be said that some of the tests were carried out intentionally using extra probing force, reaching values even higher than 10 N. However, when measuring was undertaken carefully or under controlled conditions, the common forces were seldom higher than 6 N. On the contrary, probing forces below 0.5 N usually caused probing slips, making bad contact and producing a false measurement value. To prevent this slip effect, it is always preferable to increase the force.

**Figure 12:**
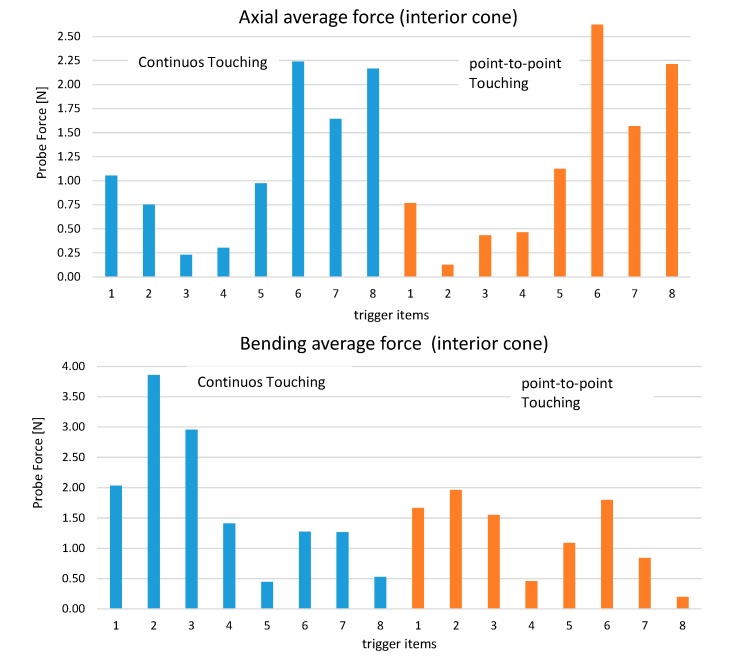
Average values of force for cones. (**Top**) axial; (**Bottom**) bending.

Last, the average forces for the operator and the feature were calculated (axial and bending). These values were calculated as the average value of force when the triggering signal was activated. For instance, Figure 12 shows the average values for the measurement of an internal cone. This feature was selected because the contribution of the two forces is more relevant. Although both forces seem to be similar in size, bending force has a stronger impact on the measurement error since its deformation is around 10 times more than in the case of axial forces. As a result, point-to-point measurement is preferred for this type of feature.

## 7. Conclusions and Future Work

According to the results, contact force is clearly a factor that should be considered and controlled in CMA performance. In this work, a new force sensor fully integrated into a CMA has been designed and manufactured. The sensor has an ergonomic design adapted to the common use of the CMA. It contains a system to acquire the bending and axial forces on the probe at the same time as the triggering signal. Forces are measured by strain gauges that measure the deformation on the probe.

The processing of the data by an application allows the synchronization of the forces and the triggering signal. The sensor was previously calibrated and subsequently validated by measuring different features. The goal of improving the probing technique was achieved, mainly in better knowledge of the force and the possibility of evaluating the two different strategies, continuous and point-to-point measurement.

The methodology of this new sensor will allow actions to be established to avoid excess force or to control several measurement parameters (such as maximum force, average force or elapsed probing time), determining good practices or compensating for contact force to improve CMA performance. Furthermore, thanks to the new sensor, probe deformation compensation models could be integrated either into the kinematic model or by directly modifying the point coordinates. This can open up new ways of increasing the accuracy.
